# Effects of different anesthetic depth during propofol anesthesia on postoperative recovery 24 h after arthroscopic day surgery: A randomized clinical trial

**DOI:** 10.3389/fphar.2022.972793

**Published:** 2022-09-16

**Authors:** Meng Ning, Yue Sun, Hao Zhang, Caiyun Chen, Linglu Sun, Lijian Chen, Zhengyuan Xia, Yao Lu

**Affiliations:** ^1^ Department of Anesthesiology, The First Affiliated Hospital of Anhui Medical University, Hefei, Anhui, China; ^2^ State Key Laboratory of Pharmaceutical Biotechnology, The University of Hong Kong, Pokfulam, Hong Kong SAR, China; ^3^ Department of Anesthesiology, Affiliated Hospital of Guangdong Medical University, Zhanjiang, Guangdong, China; ^4^ Ambulatory Surgery Center, The First Affiliated Hospital of Anhui Medical University, Hefei, Anhui, China

**Keywords:** bispectral index, anesthesia, arthroscopic, ambulatory, quality of recovery

## Abstract

**Background:** This study aimed to compare the effects of different depths of sedation during propofol anesthesia on postoperative recovery 24 h after knee arthroscopy day surgery in adult patients.

**Methods:** This prospective randomized controlled trial involved 126 patients (ASA physical status 1–2) who were scheduled to undergo arthroscopic day surgery. Patients were randomly divided into two groups: the light-sedation (L-Group) or deep-sedation (D-Group). In the L-group, the bispectral index values were kept in the range of 50–59; in the D-group, the bispectral index values were maintained in the range of 40–49. The Quality of Recovery-15 (QoR-15) score assessed 24 h postoperatively using a 15-item questionnaire was the primary outcome. Secondary outcomes included Athens Insomnia Scale scores, postoperative pain scores, nausea or vomiting.

**Results:** The total QoR-15 score 24 h postoperatively was similar in the two groups (L-group median:130, IQR [127–132] vs*.* D-group median:131, IQR [126–135], *p* = 0.089). But among the five dimensions of the QoR-15, physiological comfort was significantly better in the D-group than L-group (*p* < 0.001). The time to open eyes (*p* < 0.001), follow the command (*p* < 0.001) and to extubation (*p* < 0.001) after surgery in the L-group were shorter than the D-group. The Athens Insomnia Scale scores (*p* < 0.001) and incidence of dreaming (*p* = 0.041) at the first postoperative night in the L-group was significantly higher than those in the D-group. Propofol consumption in the L-group was less than D-group (*p* < 0.001).

**Conclusion:** For patients undergoing arthroscopic day surgery, general anesthesia with high-bispectral-index (50–59) cannot improve the total QoR-15 score 24 h postoperatively after surgery, but can lessen propofol consumption, reduce the time of extubation and anesthesia recovery period, compared with low-bispectral-index (40–49). Patients exposed to general anesthesia with low-bispectral-index values (40–49) may have better quality sleep and physical comfort than those with high-bispectral-index values (50–59).

**Clinical Trial Registration:** http://www.chictr.org.cn/showproj.aspx?proj=126526, identifier ChiCTR2100046340

## Introduction

Early recovery after surgery under general anesthesia predicts early discharge. Recovery from general anesthesia is a critical perioperative period, and plays an important role in the promotion of the effect of clinical surgical treatment from the perspective of both physiological stability and patient satisfaction (Kehlet and Dahl, 2003). Intraoperative depth monitoring of anesthesia is crucial to ensure a rapid revive and functional recovery of patients postoperatively. The bispectral index has been recognized as one of the most commonly used indicators to judge the level of sedation and depth of anesthesia, and enable the doctors to properly adjust the anesthetic dose and avoid intraoperative awareness (Gan et al., 1997; Myles et al., 2004). Studies have revealed that deep anesthesia can increase the long-term postoperative mortality of patients who undergo major surgery (Liu et al., 2019; Short et al., 2019). However, there are few studies on the effects of the depth of anesthesia on short-term postoperative functional recovery during day surgery ambulatory. Bispectral index values between 40 and 60 are optimal for depth of sedation, which can avoid intraoperative awareness and delay of wake up (Avidan et al., 2008; Chiang et al., 2018). However, the range of best depth sedation is relatively wide.

Therefore, we conducted a randomized controlled trial to compare the effects of different depths of anesthesia on postoperative recovery of patients who underwent daytime knee arthroscopy. We hypothesized that the quality of recovery scores 24 h postoperatively of light-sedation (bispectral index: 50–59) was superior to deep-sedation (bispectral index: 40–49) after knee arthroscopy day surgery. Assessing the improvement of interventions on patient experience after anesthesia and surgery requires an emphasis on patient-centered outcome measures. The quality of recovery-15 scale was selected in this study to assess recovery in five dimensions 24 h after surgery (emotional state, physical comfort, psychological support, physical independence, and pain) (Bowyer and Royse, 2016).

## Materials and methods

### Study design and study population

The trial was approval from the Ethics Committee of the First Affiliated Hospital of Anhui Medical University (Ethical Application Reference: PJ 2021-06-09 Anhui, China) and was registered at the Chinese Clinical Trial Registry (ChiCTR2100046340) on 14 May 2021, http://www.chictr.org.cn/showproj.aspx?proj=126526. In this trial, patients aged 18–65 years with ASA I–II, who were scheduled to undergo arthroscopic day surgery under general anesthesia (GA) from June 2021 to September 2021, were enrolled. The exclusion criteria were severe cardiopulmonary system diseases, endocrine system diseases: pituitary tumors, severe diabetes, pheochromocytoma, and other mental diseases, including schizophrenia, depression, alcoholism, opioid dependence; Parkinson’s disease, Alzheimer’s disease, severe insomnia, and inability to understand visual analog scale and quality of recovery-15, cases in which patients were unable to take care of themselves in their preoperative lives, hemorrhagic disease history, or abnormal coagulation function.

### Randomization

Before surgery, the researchers recruited the patients and obtained written informed consent. All the included patients were randomly divided in two groups at a 1:1 proportion using computer-generated randomization: L-group (bispectral index: 50–59) and D-group (bispectral index: 40–49). The numbers for allocation were packaged in opaque envelopes, which could only be observed by the anesthesia providers. Randomization was done on the morning of surgery using a computer-generated randomization table (simple randomization without restrictions). During a preanesthetic visit to the inpatient ward before surgery, the patients were asked to familiarize with the quality of recovery-15 questionnaire. The patients, outcome evaluators, and data information analysts were blinded to the trial intervention.

### Anesthetic procedure and intervention

Standardized monitoring processes were conducted during anesthesia and operation. Before anesthesia induction, the patients were assessed by the quality of recovery-15 questionnaire (Stark et al., 2013; Bu et al., 2016). GA was then induced by intravenous injection of sufentanil (0.4 μg kg^−1^), propofol (2.0 mg kg^−1^), and cisatracurium (0.2 mg kg^−1^). After attaining a sufficient depth of anesthesia, an I-gel laryngeal mask was utilized according to the patient’s body weight (size 3 for weights <50 kg, size 4 for 50–70 kg, or size 5 for weights >70 kg). An anesthesiologist with more than 5 years of experience was arranged to intubate the patients. All operations were performed by one surgical team.

Anesthesia was maintained using remifentanil (0.02–0.5 μg kg^−1^ min^−1^), propofol (4–8 mg kg^−1^ h^−1^), and cisatracurium (0.02–0.05 mg kg^−1^ h^−1^). The bispectral index was monitored in two groups. In the L-group, the bispectral index values were kept in the range of 50–59; in the D-group, the bispectral index values were maintained in the range of 40–49. The criteria to trigger intervention to adjust the dosage of propofol to bring back the BIS into the target range was set as the BIS index being out of the targeted range for 30 s. And, the maintenance time of BIS index of targeted range was recorded. The end-tidal CO_2_ (EtCO_2_) was kept between 35 and 45 mmHg. Patients were given 6–8 ml/kg of Ringer’s lactate solution as early as the induction period, followed by continuous infusion of Ringer’s lactate solution at a rate of 5–7 ml kg^−1^ h^−1^ until the end of surgery. Intraoperative heart rate (HR) was maintained at 50–90 beats per min; if HR < 50 beats/min, atropine (0.3–0.5 mg) was administered; if HR > 90 beats/min, esmolol was administered (0.3–0.6 mg kg^−1^. If the systolic blood pressure increased or dropped by 20% more than the baseline, nicardipine (5–10 μg kg^−1^) and ephedrine (3 mg) was given. The infusion of anesthetic drugs did not stop until the end of surgery. Approximately 15 min before the end of subcuticular closure, the anesthesiologist intravenously injected 5 µg of sufentanil for the postoperative analgesia. Ondansetron, 0.1 mg/kg, was used for antiemetic prophylaxis. At the end of the surgery, the surgeon injected 10 ml of 0.5% ropivacaine into the joint cavity for postoperative analgesia. After the operation, all patients were transported to a postanesthesia care unit. An I-gel laryngeal mask was removed by the anesthesiologists and nurse who were blinded, when the EtCO_2_ was below 45 mmHg on spontaneous respiration, and when the patient was able to follow voice commands. Flurbiprofen (50 mg) was given intravenously when the VAS score was above 3 during the postoperative period.

### Outcome measures

In this study, the primary outcome was the global quality of recovery-15 score assessed 24 h postoperatively in five dimensions: emotional state (4 items), physical comfort (5 items), psychological support (2 items), physical independence (2 items), and pain (2 items) (Bowyer and Royse, 2016). The total score on the QoR-15 ranges from 0 (the poorest quality of recovery) to 150 (the best quality of recovery). By contrast, the secondary outcome was the time to open eyes, follow voice command and extubation, hospital stays, hospitalization costs (cost from discharge to admission), and postoperative pain scores. We defined the time to open the eye as the time from the end of surgery to the opening of the eyes. Time to follow the voice commands was defined as the time from the end of surgery to the time patients responded as instructed. Additionally, the time of extubation was defined as the time from the end of surgery to removal of I-gel laryngeal masks. After surgery, the patients were asked by investigators to rate the pain of incision at 1, 6, and 24 h postoperatively using the visual analog scale (VAS) (0 = none, 10 = most severe), the Ramsay Sedation Scale (RSS) scores, the condition of sleep on the first night and postoperative nausea and vomiting (PONV) were also recorded. The incidences of awareness and dreaming was followed up on the first postoperative day. The aforementioned parameters were evaluated by the same doctor who was blinded to the different patient groups. In addition, mean arterial pressure (MAP) and HR were noted down at different time points: baseline, 5 min after intubation, 5 min after tourniquet start and release, end of surgery and extubation.

### Sample size estimation and statistical analysis

The primary outcome measure was the global quality of recover-15 score. We selected this score as the scale of sample size evaluation. According to our preliminary study conducted under GA with bispectral index values 40–49, the quality of recovery-15 scores postoperatively (at 24 h) were equivalent to 128 (12.5). In the published data, a change of 8 for the quality of recovery-15 scores was identified as clinically significant (Myles et al., 2016). We hypothesized that this trial would have 90% power to detect an increment of 8 in the quality of recovery-15 scores at a significance threshold of 0.05. Furthermore, the Power Analysis and Sample Size software (version 15.0, NCSS, LLC, United States) calculated that 53 patients per group were required. Considering a 20% withdrawal rate, we included 63 patients in each group.

Data were collected and recorded and analyzed using the Statistical Package for Social Sciences software (version 22.0, IBM Corporation, United States). The normality of quantitative variables was assessed with the Shapiro–Wilk test. Categorical variables were expressed as a number (n) and percentage (%). The quantitative variables were expressed as mean (SD), median [IQR], median (range). The mean values of age, weight, height, BMI, duration of surgery and anesthesia times were analyzed using the independent-samples *t*-test. The QoR-15 score, perioperative cumulative anesthetic dosage, time to open eyes, follow voice command and extubation, AIS scores, hospital stays, hospitalization costs were analyzed by the Mann–Whitney U-tests. The effects of intervention over time for the outcomes of interest (postoperative pain scores and hemodynamic values) were assessed using the repeated-measures analysis of variance (ANOVA) model group by time interaction. For measures that indicated significant group by time interaction effects, post hoc-analysis on differences between the two groups were assessed by the independent sample *t*-test with Bonferroni correction. The Chi-squared test or Fisher’s exact test was used to compare the number of patients based on the dream, PONV, and the ASA classification rates. Two-sided *p*-values of less than 0.05 were utilized to denote statistical significance.

## Results

**FIGURE 1:**
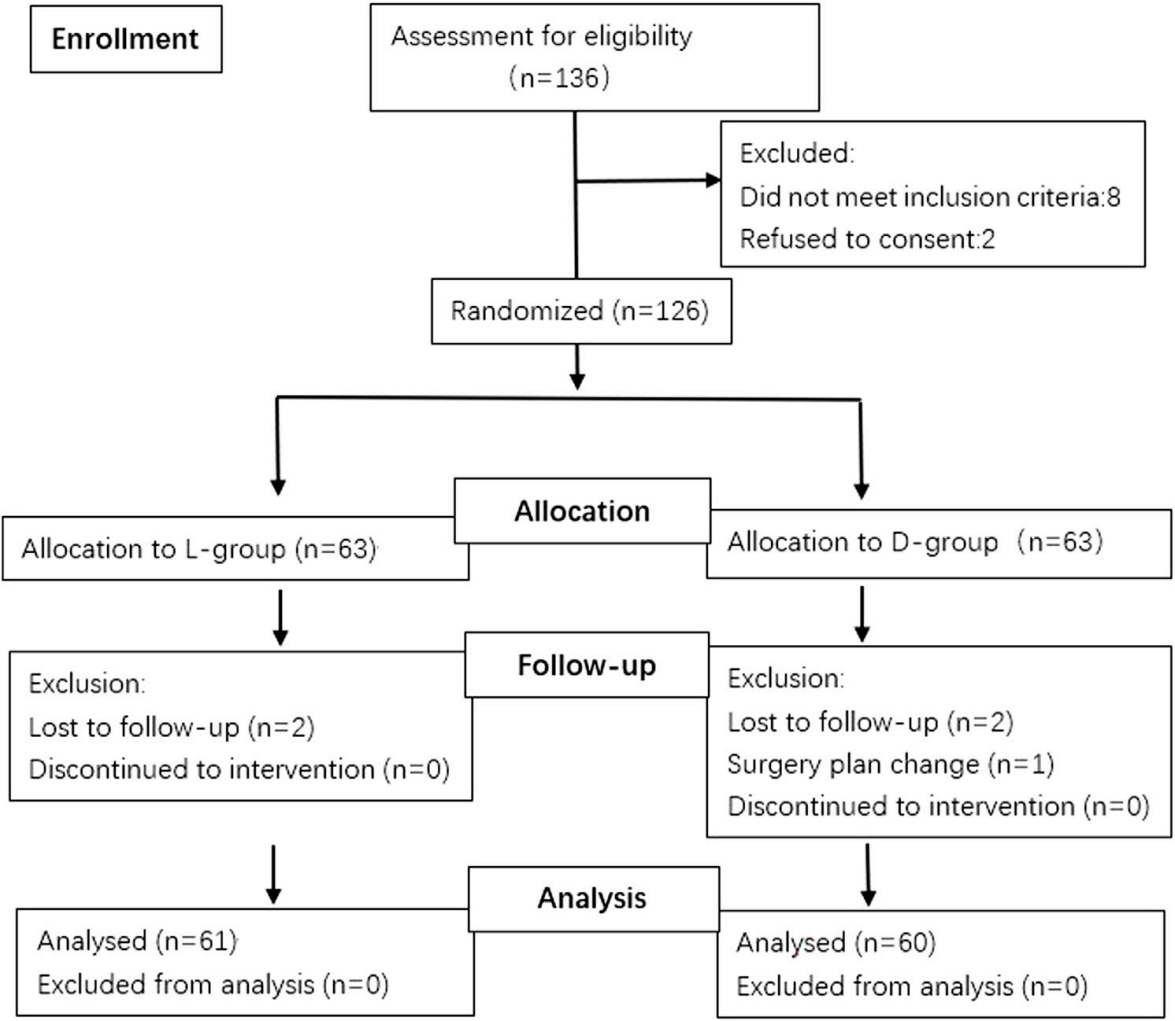
Consort flow chart that outlines patients’ assignment and treatment protocols. Patients were allocated into two groups (L-group, D-group) to receive different depths of sedation with bispectral index value maintaining in the range of 50–59 or 40–49 respectively, following a computer-generated randomization code.

**FIGURE 2:**
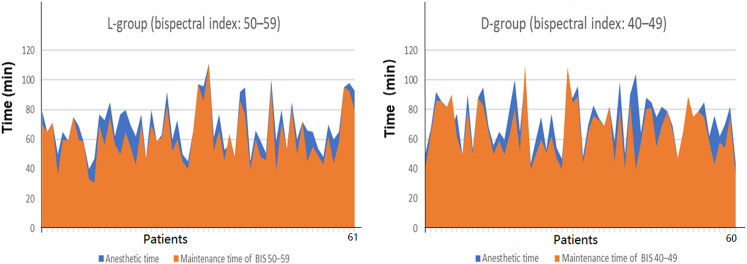
Percent of the time maintained in the target BIS values range and anesthetic time. Intraoperative maintenance time of low-bispectral-index values (40–49) and high-bispectral-index value (50–59) was insignificant (*p* = 0.163). Notes: *X*-axis in stands for patients in each group. The area of the orange range represents the time of target bispectral-index values range. The area of the blue range represents the anesthetic time.

A total of 136 patients were screened for this study from 1 June 2021, to 1 September 2021. In addition, two patients refused to consent and eight did not meet the inclusion criteria, leaving 126 for primary randomized: 63 patients in the L and 63 in the D groups. Among the randomized patients, 4 were lost to follow-up because of study withdrawal after surgery, and 1 had changed surgery plan. Thus, 121 patients were remained for the final analysis: 61 patients in the L-group, 60 in the D-group (Figure 1). The patients’ demographic profiles were comparable between the two groups (Table 1). No differences in age, gender, body mass index (BMI), ASA classification and basic bispectral index value were observed between two groups. The perioperative profiles of the patients, such as operative, anesthetic time, time of maintenance with target bispectral index values range, vasoactive drug consumption (ephedrine, atropine), the preoperative quality of recover-15 scores and RSS scores, had no significant differences between both groups (Tables 1, 2; Figure 2). However, significant differences in time to eye opening (*p* < 0.001), follow the voice command (*p* < 0.001) and extubation time (*p <* 0.001) were observed between the L and D groups. There were no patients who reported intraoperative awareness.

**TABLE.1:** Baseline characteristics of included patients in the study.

	Group L (n = 61)	Group D (n = 60)	*p*-value
Age (yr)			
Mean ± SD	45 ± 11	42 ± 13	0.188
Range	18–63	18–59
Sex, n (%)			0.524
Female	32 (52.5%)	28 (46.7%)
Male	29 (47.5%)	32 (53.3%)
BMI (kg/m^2^)	24.1 ± 2.6	23.9 ± 2.6	0.751
ASA classification, n (%)			0.792
I	14 (23.0%)	15 (25.0%)	
II	47 (77.0%)	45 (75.0%)	
Basic BIS value	96 ± 1.5	96 ± 1.9	0.702
Operative time (min)	43.7 ± 12.8	46.2 ± 15.3	0.338
Anesthetic time (min)	68.9 ± 12.4	72.8 ± 16.0	0.186
Remifentanil consumption (ug)	500 [385–675]	573 [429–676]	0.147
Sufentanil consumption (ug)	30 [30–35)	32 [30–35]	0.815
Propofol consumption (mg)	346 [250–429]	412 [359–600]	<**0.001** ^#^

AbbreviationsBMI, body mass index; ASA, american society of anesthesiologists; BIS, Bispectral index. The values are expressed as means ± SD, median [interquartile range] or number of patients (percentage). #*p* < 0.05.

**TABLE.2:** Perioperative profiles of the patients.

	Group L (n = 61)	Group D (n = 60)	*p*-value
Maintenance time of target BIS range (min)	62 ± 17	66 ± 17	0.163
Time to open eyes (min)	6 [5–8]	9 [8–11]	<**0.001** ^#^
Time to follow the command after surgery (min)	7 [6–9]	11 [9–13]	<**0.001** ^#^
Time to extubation (min)	9 [8–10]	12 [10–14]	<**0.001** ^#^
Atropine (mg)	0 (0–0.5)	0 (0–0.5)	0.388
Ephedrine consumption (mg)	6 [0–12]	8 [0–12]	0.563
RSS	2 [1–3]	2 [2–4]	0.085
Intraoperative awareness	0	0	NA
Hospital Stay (h)	23 (17–48)	23 (21–48)	0.609
Hospitalization costs (¥)	12,263 [12,016–12502]	12,355 [11,999–12850]	0.332
AIS scores at the first postoperative night	4 [3–6]	2 [1–3]	<**0.001** ^#^
Patients having dream, n (%)			**0.041** ^#^
Yes	16 (26%)	7 (12%)	
No	45 (74%)	53 (88%)	
Postoperative VAS score			0.127
1 h	0 (0–4)	1 (0–4)	
6 h	1 (0–4)	1 (0–6)	
24 h	1 (0–4)	1 (0–4)	
PONV, n (%)			0.131
Yes	9 (15%)	4 (7%)	
No	52 (85%)	56 (93%)	

Abbreviations BIS, bispectral index; RSS, ramsay sedation scale; VAS, visual analogue scale; AIS, athens insomnia scale; PONV, postoperative nausea and/or vomiting. The values are expressed as means ± SD, median (interquartile range[range]), median (range) or number of patients (percentage). #*p* < 0.05.

No differences in the total QoR-15 scores 24 h postoperatively were observed in the L-group (*p* = 0.089, Table 3). But among the five dimensions of the QoR-15, physiological comfort was significantly better in the D-group than L-group (*p* < 0.001, 48 [46–40] vs*.* 46 [45–47.5]). The time to eye opening, follow voice command, and extubation in the group were shorter than the D-group (6 [5 to 8] vs. 9 [8 to 11] min, *p* < 0.001; 7 [6 to 9] vs. 11 [9 to 13] min*, p* < 0.001; 9 [8 to 10] vs. 12 [10 to 14] min*, p* < 0.001, respectively, Table 2). The Athens Insomnia Scale scores (*p* < 0.001) and incidence of dreaming (*p* = 0.041) at the first postoperative night in the L-group was significantly higher than the D-group (4 [3 to 6] vs. 2 [1 to 3], *p* < 0.001; 26 vs 12%, *p =* 0.041, Table 2). Propofol consumption in the L-group was less than the D-group (*p* < 0.001, Table 1).

**TABLE 3:** The QoR-15 scores (121 patients) before surgery and 24 h after surgery between two groups.

	Group L (n = 61)	Group D (n = 60)	*p*-value
Preoperative score			
Physical comfort	49 [46–49]	48.5 [47–49]	0.870
Physical independence	20 [20–20]	20 [20–20]	0.193
Pain	15 [14.5–16]	16 [15–17]	0.313
Psychological support	20 [20–20]	20 [20–20]	0.516
Emotional state	39 [37.5–40]	39 [38–40]	0.858
Total QoR-15 score	142 [139–144]	142.5 [140–145]	0.279
Postoperative score			
Physical comfort	46 [45–47.5]	48 [46–49]	<**0.001** ^#^
Physical independence	7 [7–7]	7 [7–7]	0.344
Pain	18 [17–18.5]	18 [17–19]	0.958
Psychological support	20 [20–20]	20 [20–20]	0.135
Emotional state	39 [38–39.5]	39 [38–40]	0.155
Total QoR-15 score	130 [127–132]	131 [126–135]	0.089

AbbreviationsQoR-15, quality of recovery-15; The values are expressed as means ± SD, or median [interquartile range]. #*p* < 0.05.

**FIGURE 3:**
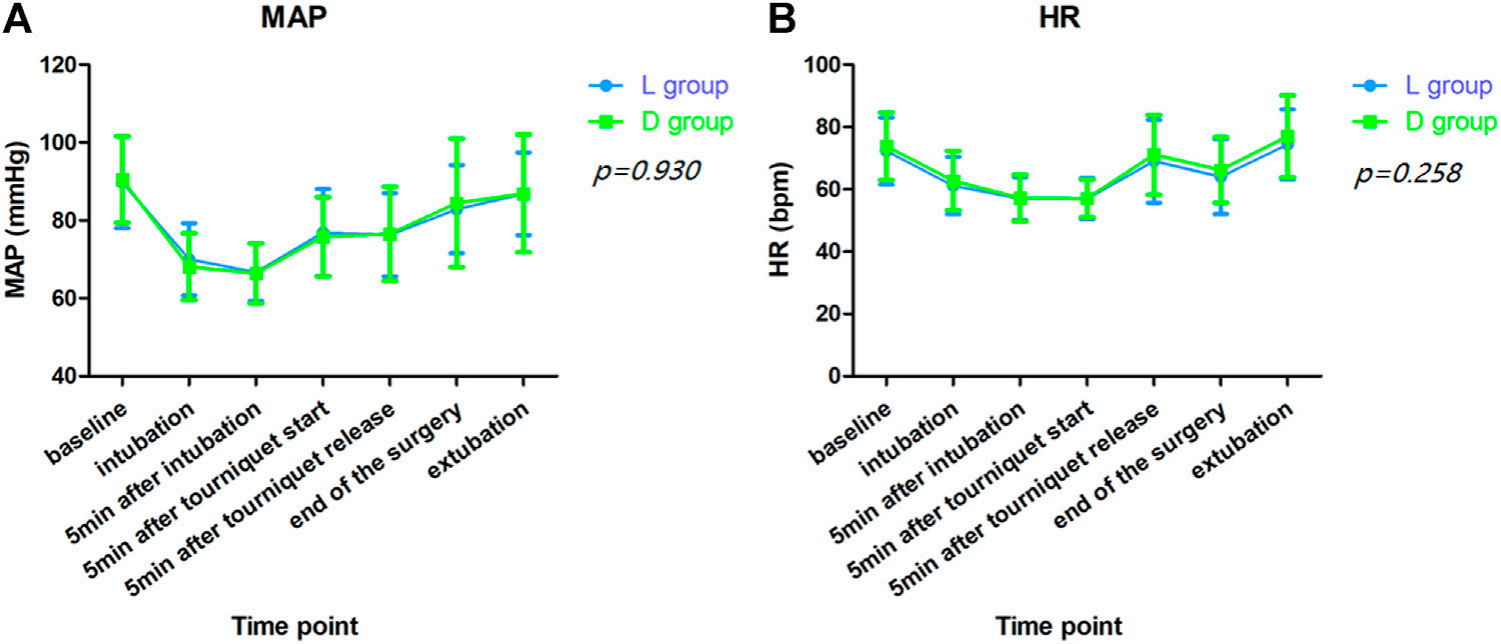
Hemodynamic values. **(A)**. Mean artery pressure (MAP); **(B)**. Heart rate (HR). No significant differences were observed in MAP (*p* = 0.930) and HR (*p* = 0.258) at baseline, 5 min after intubation, 5 min after tourniquet onset and release, end of surgery and extubation between both groups by repeated measures analysis of variance.

Hemodynamic profiles, such as HR and MAP, were compared between the two groups. No significant differences were observed in MAP at baseline, 5 min after intubation, 5 min after tourniquet onset and release, end of surgery and extubation between both groups (Figure 3). Furthermore, perioperative opioid consumption (sufentanil, remifentanil), postoperative visual analog scale for incision site pain between the two groups were not significantly different and the difference in the incidence of PONV between the both groups was also insignificant (Tables 1, 2).

## Discussion

The main findings of this study indicated that compared with the D-group (bispectral index: 40–49), GA for patients undergoing knee arthroscopy day surgery (with bispectral index values in the range of 50–59) did not improve the total QoR-15 score 24 h postoperatively after surgery but was able to lessen propofol consumption, the time of recovery from the anesthesia, and extubation. Furthermore, patients who were exposed to GA (with bispectral index values in the range of 40–49) have better quality sleep and physical comfort than light sedation (bispectral index: 50–59) at the first night after surgery.

Knee arthroscopy is a common clinical day surgery (Romina et al., 2017). Promoting day surgery can reduce the length of stay and enhance recovery after surgery, which could reduce the risk of venous thromboembolism and hospital-acquired infections (Bailey et al., 2019). The quality of recovery from GA can impact patient safety, patient satisfaction and medical costs (Fritz et al., 2013). Several studies had reported that bispectral index monitoring for general anesthesia may result in lower anesthetic doses, a lower incidence of anesthesia awareness, and faster patient recovery (Liu, 2004; Fritz et al., 2013; Lewis et al., 2019). In this study, propofol consumption in the L-group was less compared with the D-group, and no patients were reported intraoperative awareness. However, the total QoR-15 scores 24 h after knee arthroscopy day surgery in the L-group (bispectral index: 50–59) was not higher than the D-group (bispectral index: 49–49) (*p* > 0.05). This means that light sedation (bispectral index: 50–59) cannot improve the quality of recovery from GA 24 h postoperatively compared with deep sedation (bispectral index: 40–49). Therefore, it may not require maintaining bispectral index values in the range of 40–49 and consume more anesthetics for knee arthroscopy. McCormick et al. suggested that prolonged cumulative double-low conditions (low MAP (<75 mmHg) and low-bispectral-index values (<45)) were associated with mortality (McCormick et al., 2016). Furthermore, Yoon et al. reported that the cumulative duration of double-low conditions [low MAP (<45 mmHg) and low-bispectral-index values (<40)] were associated with 90-days postoperative mortality, and not with a 180-days postoperative mortality (Yoon et al., 2020). The appropriate dose for a given patient may contribute to the faster recovery and lower medical costs by reducing the time during the operating room and PACU (Dexter et al., 1999; Myles et al., 2004; Clark et al., 2009; Bosslet et al., 2010). In this study, light sedation (bispectral index: 50–59) reduced the time to eye opening, follow to voice command, and extubation and enhancement of the recovery from anesthesia compared with the D-group (bispectral index: 40–49), which was consistent with previous studies (Leslie et al., 2005; Mashour et al., 2012). However, no significant difference in medical costs and hospital stays was observed between the L-group (bispectral index: 50–59) and D-group (bispectral index:40–49), which means that light sedation (bispectral index: 50–59) cannot save hospitalization spending and reduce the length of stay. Additionally, there were no significance differences in hemodynamic profiles, vasoactive drug consumption (ephedrine, atropine), opioid consumption (sufentanil, remifentanil), and postoperative visual analog scale (VAS). Compared with the D-group (bispectral index: 40–49), maintaining bispectral index values at 50 to 59 may not increase opioid and vasoactive drug consumption, and may not affect the postoperative VAS and the occurrence of PONV.

High-sleep quality after surgery is one of the important guarantees for postoperative rehabilitation of patients (Rosenberg-Adamsen et al., 1996; Chen et al., 2017). Studies showed that sleep disturbance are more likely to occur after surgery owing to postoperative pain, environmental changes, trauma and other factors, and may contribute to neurological, cardiovascular complications, and may lead to increased morbidity (Redwine et al., 2000; Leung and Bradley, 2001; Alhola and Polo-Kantola, 2007; Krenk et al., 2012). Therefore, improving the sleep quality after surgery probably has a positive effect on the recovery of surgical patients. In this study, the Athens Insomnia Scale scores associated with the sleeping period of the first night after knee arthroscopic surgery in the L-group (Bis 50–59) was higher than the D-group (bispectral index: 40–49). Thus, low-bispectral index values (40–49) can improve insomnia conditioned and the quality of sleep during the first night after surgery. This phenomenon may be one reason for the better physical comfort score in the D-group (bispectral index: 40–49). Different propofol consumptions may contribute to the above phenomenon. Dinesh Pal’s findings showed that propofol could modulate sleep homeostasis by compensating for sleep debt in sleep-deprived rats, thus satisfying the need for both rapid and nonrapid eye movement sleep patterns (Pal et al., 2011). Evidence suggested that sufentanil may impair sleep and sleep architecture and insomnia may increase anesthetic consumption, but there was no difference in opioid consumption between the two groups (Erden et al., 2016; Tripathi et al., 2020; Yang et al., 2021). Increased propofol consumption in the D-group (bispectral index: 40–49) may be the possible reason for the improvement of the quality of sleep during the first night after surgery. Another positive result is that the numbers of patients reported of dreaming at first night sleep postoperatively in the D-group was less than the L-group. Combination of Athens Insomnia Scale scores, the occurrence of dreaming, and low-bispectral-index values (40–49) improved the first sleep quality by reduction in AIS scores and incidence of dreaming.

Before the study, we hypothesized that high-bispectral-index values (50–59) improves the quality of recovery scores 24 h postoperatively after knee arthroscopy day surgery, when compared to low-bispectral-index values (40–49). However, the results were contrary to our expectations. During the operation, intraoperative maintenance time of low-bispectral-index values (40–49) and high-bispectral-index value (50–59) was insignificant ([66 ± 17 vs. 62 ± 17] min, *p* = 0.163, table 2 and Figure 2). Propofol consumption in the L-group (bispectral index 50–59) was less and high-bispectral-index values (50–59) can shorten anesthesia recovery period. In addition, for patients with insomnia, low-bispectral-index values (40–49) may be more suitable. This may contribute to patients’ physical comfort score. Several studies suggested that patients with sleep disorders may benefit from operations performed in the morning and GA under a median bispectral index level of 39 may contribute to better recovery of cognitive function 4–6 weeks postoperatively compared with a median bispectral index level of 51, particularly with respect to the ability to process information (Farag et al., 2006; Song et al., 2020). The understanding of the influences of different depths of anesthesia on postoperative cognitive function requires additional research. The aforementioned facts are the reasons for the results of this trial.

This trial is associated with several limitations. First, this is only a single-center study. Thus, a multicenter study would be better for testing our hypothesis. Second, as no “gold standard” exists for the assessment of the quality of recovery after surgery and anesthesia, the quality of recovery-15, was commonly used recently for validations. More measures should be developed to assess the quality of recovery. Third, the duration of surgery and hospitalization of patients were short, and the time to observe was limited; it is difficult to compare the long-term effects on the patients. Finally, the effects of different bispectral index values on the older patients or the children are unknown.

In conclusion, this study demonstrated that in patients who undergo arthroscopic day surgery, GA with high-bispectral index values (50–59) cannot improve the total QoR-15 score 24 h postoperatively but can lessen propofol consumption, accelerate the time of anesthetic recovery compared with low-bispectral-index values (40–49). Patients exposed to GA with low-bispectral-index values (40–49) have better quality sleep and physical comfort than those with high-bispectral-index values (50–59).

## Data Availability

The original contributions presented in the study are included in the article/supplementary material, further inquiries can be directed to the corresponding authors.
